# Supplementary material for: Increased Intake of Both Caffeine and Non-Caffeine Coffee Components Is Associated with Reduced NAFLD Severity in Subjects with Type 2 Diabetes
Source: Nutrients. 2022 Dec 20;15(1):4. doi: 10.3390/nu15010004 (PMC9824649; doi:10.3390/nu15010004)
Supplement: Supplementary file 1 [file nutrients-15-00004-s001.zip › nutrients-2113236-supplementary.pdf]

Increased intake of both caffeine and non-caffeine coffee components is associated with reduced NAFLD severity in subjects with type 2 diabetes

Margarida Coelho [†], Rita S. Patarrão [†], Inês Sousa-Lima, Rogério T. Ribeiro, Maria João Meneses, Rita Andrade, Vera M. Mendes, Bruno Manadas, João Filipe Raposo, M. Paula Macedo and John G. Jones*

[†]Joint first authors
*Corresponding author

Supplementary material

Table S1. Quantities of selected caffeine and non-caffeine metabolites measured from 24 h urine collections. Values are expressed as mean ± standard deviation.

Caffeine metabolites	Daily amount (mg)	Percentage relative to total (%)
Caffeine	4.95±5.05	8.80±5.20
Paraxanthine	49.48±43.38	87.44±9.26
Theobromine	53.37±81.11	-
Theophylline	0.93±0.83	3.76±8.76
**Non-caffeine metabolites**		
Trigonelline	26.06±20.32	94.65±4.55
p-Coumaric acid	0.16±0.14	0.97±1.26
trans-Caffeic acid	1.00±0.9	4.38±3.71

Table S2: Correlations, expressed as Pearson coefficients, of individual caffeine and non-caffeine metabolites (total amount recovered in 24-hour urine) and reported espresso coffee consumption (cups per day). The *p* value for each Pearson coefficient is shown alongside in parentheses.

**Caffeine metabolites**	**Correlation with cups of espresso per day**
Caffeine	0.2859** (*p*=0.0045)
Paraxanthine	0.4301**** (*p*<0.0001)
Theophylline	0.3397*** (*p*=0.0007)
**Non-caffeine metabolites**	
Trigonelline	0.3444*** (*p*=0.0006)
p-Coumaric acid	0.08741 (*p*=0.3946)
Caffeic acid	0.4033**** (*p*<0.0001)

Table S3: Multiple regression analysis for fatty liver index (FLI) and urinary coffee metabolites (total caffeine metabolites, top table and total non-caffeine metabolites, bottom table) normalized to fat-free mass.

Dependent variable: FLI				
**Explanatory variables**	**Coefficients**	**S.E.**	**t**	***p* value**
Intercept	82.585	30.227	2.732	0.008
HbA1c	2.371	1.733	1.368	0.175
Sex	4.279	6.032	0.709	0.480
Age	-0.304	0.296	-1.029	0.307
Alcohol consumption	-0.145	0.158	-0.919	0.361
Glomerular filtration rate (GFR)	-0.184	0.193	-0.953	0.343
Total caffeine metabolites	-2.507	1.090	-2.301	0.024*
R^2	0.147			
Adjusted R^2	0.081			
F-statistic	2.233			
Significance F	0.049*			

Dependent variable: FLI				
**Explanatory variables**	**Coefficients**	**S.E.**	**t**	***p* value**
Intercept	98.660	28.909	3.413	0.001
HbA1c	2.415	1.669	1.447	0.152
Sex	-0.586	5.903	-0.099	0.921
Age	-0.339	0.284	-1.193	0.237
Alcohol consumption	-0.176	0.152	-1.155	0.251
Glomerular filtration rate (GFR)	-0.247	0.181	-1.366	0.176
Total non-caffeine metabolites	-7.057	2.091	-3.375	0.001***
R^2	0.205			
Adjusted R^2	0.144			
F-statistic	3.348			
Significance F	0.005**			

Table S4: Multiple regression analysis for fatty liver index (FLI) and urinary coffee metabolites (total caffeine metabolites, top table and total non-caffeine metabolites, bottom table), normalized to total body weight.

Dependent variable: FLI				
**Explanatory variables**	**Coefficients**	**S.E.**	**t**	***p* value**
Intercept	77.949	30.306	2.572	0.012
HbA1c	2.439	1.724	1.415	0.161
Sex	6.099	6.093	1.001	0.320
Age	-0.294	0.294	-1.000	0.320
Alcohol consumption	-0.131	0.157	-0.836	0.406
Glomerular filtration rate (GFR)	-0.167	0.193	-0.868	0.388
Total caffeine metabolites	-9.375	3.737	-2.508	0.014*
R^2	0.157			
Adjusted R^2	0.092			
F-statistic	2.415			
Significance F	0.034*			

Dependent variable: FLI				
**Explanatory variables**	**Coefficients**	**S.E.**	**t**	***p* value**
Intercept	98.284	28.586	3.438	0.001
HbA1c	2.382	1.649	1.444	0.153
Sex	1.059	5.766	0.184	0.855
Age	-0.348	0.281	-1.239	0.219
Alcohol consumption	-0.165	0.150	-1.097	0.276
Glomerular filtration rate (GFR)	-0.255	0.178	-1.429	0.157
Total non-caffeine metabolites	-28.134	7.700	-3.654	0.0005***
R^2	0.222			
Adjusted R^2	0.162			
F-statistic	3.706			
Significance F	0.003**			

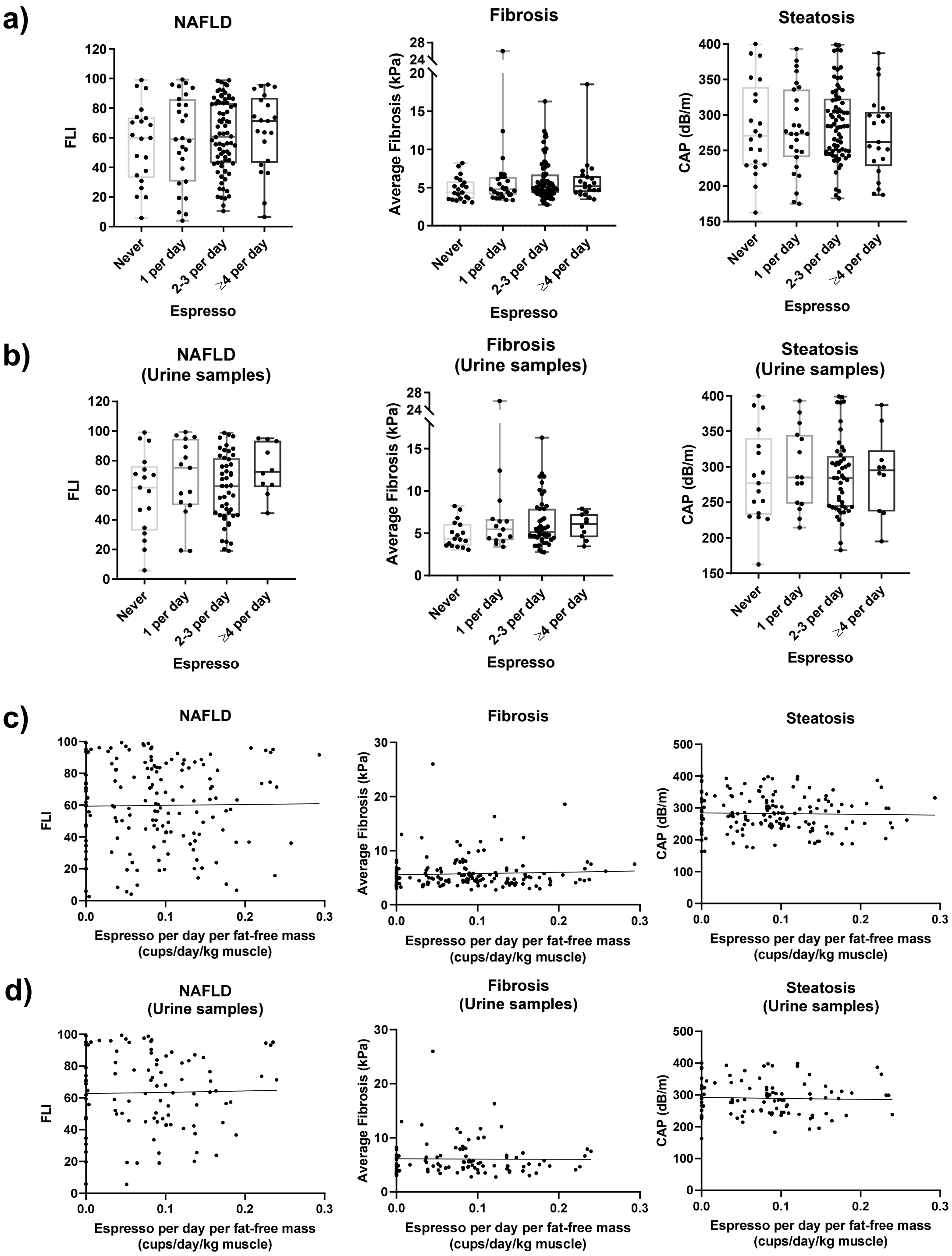

Figure S1: No significant associations were found between self-reported coffee consumption, either adjusted (**c**,**d**) or non-adjusted for fat-free mass (**a**,**b**), and any NAFLD parameter either for the cohort that provided the 24-hour urine samples (**b**,**d**) or for the entire study population (**a**,**c**). Non-significant according to Mann-Whitney test for a and b. Non-significant according to Pearson correlation for c and d.

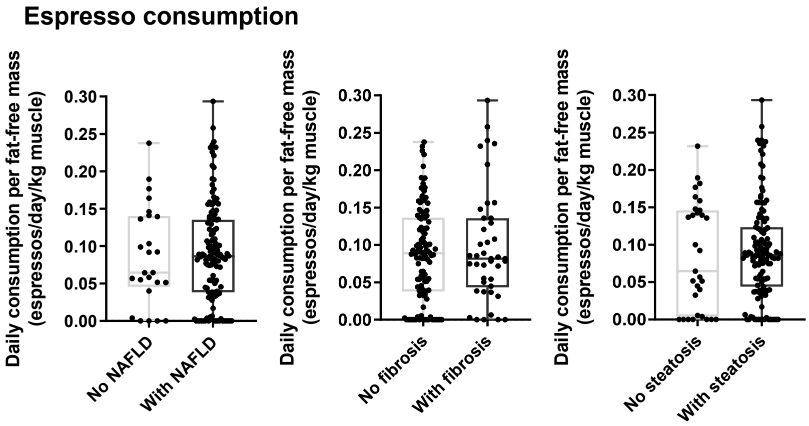

Figure S2: Levels of espresso consumption, adjusted for fat-free mass with different categories of the fatty liver index (FLI) and scores of fibrosis and steatosis obtained by Fibroscan®. Non-significant according to Mann-Whitney test.

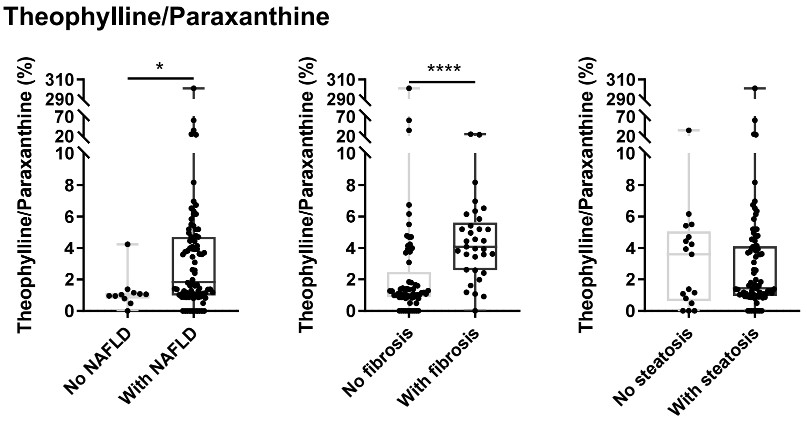

Figure S3: Ratio of theophylline abundance by paraxanthine with different categories of the fatty liver index (FLI) and scores of fibrosis and steatosis obtained by Fibroscan®. * = significant difference ($p \leq 0.05$, Mann-Whitney test); **** = significant difference ($p \leq 0.0001$, Mann-Whitney test).
